# eB4CAST Approach Improves Science Communication With Stakeholders in a College-Based Health Program

**DOI:** 10.3389/fpubh.2020.00158

**Published:** 2020-05-07

**Authors:** Melissa D. Olfert, Makenzie L. Barr, Rebecca L. Hagedorn, Rachel A. Wattick, Wenjun Zhou, Tanya M. Horacek, Anne E. Mathews, Kendra K. Kattelmann, Tandalayo Kidd, Adrienne A. White, Onikia N. Brown, Jesse Stabile Morrell, Lisa Franzen-Castle, Karla P. Shelnutt, Carol Byrd-Bredbenner, Terezie Tolar-Peterson, Geoffrey W. Greene, Sarah E. Colby

**Affiliations:** ^1^Division of Animal and Nutritional Sciences, West Virginia University, Morgantown, WV, United States; ^2^Business Analytics and Statistics, University of Tennessee, Knoxville, Knoxville, TN, United States; ^3^Department of Public Health, Food Studies, and Nutrition, Syracuse University, Syracuse, NY, United States; ^4^Food Science and Human Nutrition Department, University of Florida, Gainesville, FL, United States; ^5^HNS Department, South Dakota State University, Brookings, SD, United States; ^6^Department of Food, Nutrition, Dietetics and Health, Kansas State University, Manhattan, KS, United States; ^7^School of Food and Agriculture, University of Maine, Orono, ME, United States; ^8^Department of Nutrition, Dietetics, and Hospitality Management, Auburn University, Auburn, AL, United States; ^9^Department of Agriculture, Nutrition, and Food Systems, University of New Hampshire, Durham, NH, United States; ^10^Nutrition and Health Sciences Department, University of Nebraska–Lincoln, Lincoln, NE, United States; ^11^Department of Family, Youth and Community Sciences, University of Florida, Gainesville, FL, United States; ^12^Department of Nutritional Sciences, Rutgers University, New Brunswick, NJ, United States; ^13^Department of Food Science, Nutrition and Health Promotion, Mississippi State University, Starkville, MS, United States; ^14^Department of Nutrition and Food Science, University of Rhode Island, Kingston, RI, United States; ^15^Department of Nutrition, University of Tennessee, Knoxville, Knoxville, TN, United States

**Keywords:** dissemination, infographic, campus, communication, feedback

## Abstract

Communicating scientific results with community partners is often lacking in intervention programs, thus eB4CAST was developed to facilitate impact sharing. This article investigated using the eB4CAST dissemination tool to communicate impact from a campus-based obesity prevention program. Data from Get Fruved RCT university sites collected at baseline were used to generate eB4CAST reports. Experts (*n* = 13) and RCT sites (*n* = 15) were asked to provide feedback on eB4CAST reports based on appeal, understanding, and clarity. On all Likert items, participants rated above 7 on each (out of 10). Positive responses from open-ended questions included eB4CAST reports being clear, visually appealing, and aid in program understanding. Overall, eB4CAST was successful in relaying data and information for the Get Fruved program, thus a means for science communication that could be used in interventions. Utilizing infographics to report data and information is a feasible way to disseminate and communicate in a cost-effective, timely manner.

## Introduction

Dissemination and Implementation (D&I) research has become a priority within organizations such as the Centers for Disease Control and Prevention (CDC) and the National Institute of Health (NIH) with a strong focuses on the involvement of community members and sharing of evidence-based research (1). Therefore, this research, combined with Community Based Participatory Research (CBPR) principles, which is the collective equal efforts of both researchers and community members to developing aspects of a program for their target population, have become key aspects of lifestyle interventions in order to promote program sustainability, and longevity (2). However, despite the efforts to improve the dissemination and implementation of programming into communities, a gap remains between research driven interventions and community ownership. This calls attention to potential barriers that may play a role in the dissemination and implementation process.

Best practices for the dissemination of programming are needed for successful implementation (3). Previous research has shed light on some fundamental requirements for successful program dissemination into the community settings (4–8). One of the largest barriers to overcome when disseminating a program is effective communication (9). There is often a language barrier between academic discourse and that of the community (9). Researchers are often pressured to use traditional communication methods for findings, such as journal or conference publications, which are not commonly viewed by the community. Further, the technical nature of scientific writing can cause confusion with a lay population. This communication divide may result in a community population that does not understand the value of the program and therefore small, if any, program dissemination occurs at the community level (10, 11). In the dissemination realm, science communication is “a matter of transmitting information about science from scientific experts to the public (12).”

Use of ineffective communication methods also causes researchers to fail in building a partnership that translates to ownership among decision-making stakeholder to gain support for program continuation. Stakeholders are defined as “people with a vested interest in a particular outcome” (13) and in the context of this paper are the institutional leaders that disseminate interventions on college campuses. Partnership with campus stakeholders are essential for program success (5), thus ensuring that campus communities understand the value and impact of a program is key. Utilization of CBPR principles can aid in overcoming these barriers by working to provide communities ownership of the data to share the impact that is meaningful for the target population (9). CBPR allows for the development of collaborative relationships by understanding and promoting community priorities (14, 15). To accomplish this, researchers should provide program outcomes back to the community and justification for support from stakeholders for program dissemination.

Finding effective tools that utilize CBPR principles to make D&I science easier and more feasible for community members is essential. Recently a dissemination tool, eB4CAST, that merges D&I and CBPR principles, has been developed to assist community-based programs in communicating program outcomes and gaining community and stakeholder support. The eB4CAST framework was designed to provide community members with an evidence-based forecast to capture (C), assemble (A), sustain (S) and present the timelessness (T) of program/research data (16) and is a published and tested tool (16, 17) that uses infographic reports to share data back with the community in an easily understood manner. Infographics are used as visual guides to quickly share data in a concise way that general audiences can understand (18–20). These infographic reports can show complex data digitally and visually and have been promoted as a means to easily disseminate a community program in an understandable manner (17). Additionally, digital formats are more cost-effective than traditional measures, such as printed copies of dissemination, which is beneficial to communities with limited resources (17, 21, 22). The eB4CAST reports are tailored for individual communities and versatile for distribution from eB4CAST researchers directly to participants, stakeholders, and future potential leaders of a program's dissemination. Through eB4CAST, data is given back to communities and stakeholders so that they can easily demonstrate the impact and feasibility of programs, thus overcoming the aforementioned barriers to program dissemination.

The overarching goal of eB4CAST is for the report to be utilized by community members and stakeholders to facilitate communication, be a low-cost option for dissemination, and show program impact. To date, eB4CAST has been used in a youth and adult childhood obesity prevention program (17) but was developed to be used in diverse community-based programs. Thus, as each project utilizes its own unique data points and variables to capture information, reports are also tailored to each project from a graphic design standpoint. Therefore, as intervention programs use the eB4CAST dissemination tool, it is necessary to continue testing and refining the infographic reports for each unique program to ensure the reports meet the intended outcomes. For this study, eB4CAST reports were created for the Get Fruved (short for FRUits and VEgetables) intervention. The Get Fruved eB4CAST infographics were intended to provide data back to sites and participants to facilitate discussion with campus stakeholders and provide the framework for future programming efforts. The purpose of the study was to obtain feedback from content experts, and from Get Fruved randomized control trial study site leads, regarding the clarity, usefulness, and appeal of eB4CAST reports that were generated from the Get Fruved data collected at each participating school.

## Materials and Methods

**Figure 1:**
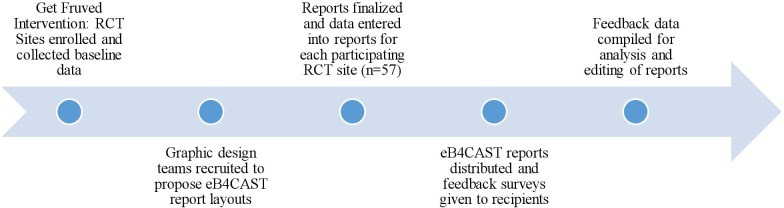
Overview of eB4CAST process in Get Fruved randomized control trial.

The overarching study, Get Fruved, is a multi-state, peer-led, social marketing, and environmental change obesity prevention campaign for college students funded by the United States Department of Agriculture. The Get Fruved intervention was a 5-year program that began in 2014. As Get Fruved was a large multi-state program, lead researchers wanted to supply those sites who enrolled in Get Fruved with their own data and feedback from the intervention. To supply this data in an easy-to-read format, eB4CAST was employed due to its systematic method to dissemination data to multiple sites (16). The eB4CAST reports were generated during the randomized-control trial (RCT) phase of Get Fruved in 2017–2018. Universities across the U.S. submitted mini-grant applications to be a part of the RCT, and ultimately, 62 higher education institutions participated. Higher education institutions were divided into 6 groups based on geographical location (Southeast, Northeast, Northcentral, Midwest, Northwest, Southwest) and matching pairs were created based on geographic region, type of organization (Historically Black Colleges and Universities, Hispanic Serving Institutions, Tribal College/University, community college, and 4-year institutions), size organization, public/private, and race/ethnicity profile of the institution. From pairs, random selection was used to designate one site selected to be an intervention site resulting in 31 intervention sites and 32 control sites. An overview of the eB4CAST implementation in Get Fruved is depicted in Figure 1.

The multi-state umbrella Institutional Review Board (IRB) at the West Virginia University approved the study for all universities designing eB4CAST and all Get Fruved RCT sites (IRB approval #14-09366 B-XP). This study was prospectively registered in October 2016 on clinicaltrials.gov, NCT02941497.

Intervention sites were asked to collect both direct and indirect data. Indirect data included demographics of the city population, student population, race/ethnicity of students, health resources and facilities on campus, student-teacher ratio, and percentage of students in-state vs. out-of-state. All indirect data was collected via the RCT institution website and national websites including countyhealthrankings.org and ers.usda.gov. Direct data were obtained from all individual students enrolled in the Get Fruved program at baseline and post intervention, self-reporting their own demographics, self-reported health behaviors from four validated tools to capture dietary intake [National Cancer Institute's Fruit and Vegetable screener (23)], physical activity levels [International Physical Activity Questionnaire (24)], stress levels [Perceived Stress Scale (25)], and sleep quality [Pittsburgh Sleep Quality Index (26)] to develop a “Wellness Report” for each site. These survey tools from the Get Fruved study have been described previously (27). Information from the “Wellness Report” and four other researcher-designed tools were included in the eB4CAST reports. The four developed tools were the Health Campus Environmental Audit (HCEA) (28–33), the College Environmental Perceptions Survey (CEPS) (34), Student and Administrative Readiness to Change Survey, and Student and Administrative Priorities Survey (Table 1) (35).

**Table 1:** Survey tools used in the eB4CAST reports and description of their measures.

**Tools**	**Description**
Indirect data	City population, student population, race/ethnicity of students, health resources and facilities on campus, student-teacher ratio, and percentage of students in-state vs. out-of-state.
Health Campus Environmental Audit (HCEA) (28–33)	Examines the campus environment in areas including: healthfulness of dining halls on campus; campus policies regarding health; and quality and availability of recreational services.
College Environmental Perceptions Survey (CEPS) (34)	Determines the student perceptions of their environment in the areas of Policy, Food, Water, Vending, Physical Activity, Stress, and Sleep.
Student and administrative readiness to change survey	Determines the personal readiness to make health changes on campus by both student populations and administrative populations.
Student and administrative priorities survey	Determines the most important concepts on campus by ranking (i.e., safe, clean, available water on campus).
Wellness report card (23–26, 35)	Each site received wellness reports that included the National Cancer Institute's fruit and vegetable screener, the international physical activity questionnaire, the perceived stress scale, and the pittsburgh sleep quality index.

The design process for eB4CAST dissemination reports is handled by the eB4CAST research team to prevent burden on program leaders and is customized for different community-based programs, as outlined previously (16). To design eB4CAST reports specific for the GetFruved program, informational emails were sent to 16 graphic design experts across the nation to initiate conversations of interest in developing an ideal report to disseminate program findings to stakeholders and community members. From these conversations, three teams of graphic designers were employed to build their own proposal design to the best visual reporting system. Teams included a graphic design student, a university-employed graphic designer, and a freelance graphic designer. Design teams were given mock data from Phase I data collection to design their reports. This method was used solely for design purposes to ensure the final report was visually and graphically appealing created by expert graphic design staff. Upon completion of each graphic design team eB4CAST report, aspects of each design were chosen and compiled by the lead research team at West Virginia University (researchers on both eB4CAST design and utilization as well as Get Fruved) to build a final comprehensive report. This final eB4CAST report was ultimately designed to be given to stakeholders at each Get Fruved RCT sites at baseline and again post-intervention (reflecting changes related to the program implementation). Reports were given to sites with their school's baseline data included (data included survey results and HCEA reports; Table 1). Get Fruved sites were asked to utilize their eB4CAST reports to promote the program on their campus, engage students in discussions about health-related issues and priorities, communicate with the administration to advocate for environmental changes to support health and communicate their work. As the purpose of reports was to be used for a variety of individuals, reports were tailored to be easy-to-read and clear for all users.

Feedback from content experts completed a survey to evaluate the eB4CAST reports. Experts were selected based on previous knowledge of Get Fruved, CBPR and/or Reach, Effectiveness, Adoption, Implementation, Maintenance [RE-AIM] framework science. Experts were recruited via email from the multi-state that developed the Get Fruved program, the Healthy Campus Research Consortium, which is comprised of researchers focused on developing interventions for college aged populations. Content expert feedback was incorporated prior to finalization of the reports being sent to each RCT site. An 18-item survey was sent through email to researchers along with a copy of the draft eB4CAST report. Survey questions, adapted from previous research on the application of eB4CAST in another obesity prevention program, iCook 4-H (16), addressed visual appeal, usage of the content, understandability of the data, and addressing grammatical errors on a scale of 1 being strongly disagreed to 10 strongly agreeing. Participants were further asked to address if any information was not included that may be helpful. Feedback was compiled and addressed to make changes to the report prior to finalizing the eB4CAST post-intervention reports. Summative content analysis was used to compile the short feedback comments and quotes (36).

eB4CAST reports were given to intervention sites at baseline (Forecast report; December 2017). Stakeholders and site leaders at each Get Fruved RCT intervention site (faculty members, wellness program leaders, or registered dietitians) received an electronic version of the report, a PowerPoint presentation with notes, a poster to hang on their campus to show involvement, and 25 hard copies of their report. Control sites were given reports that included baseline data in May of 2018. When RCT site stakeholders and site leaders received their electronic and mail packages of eB4CAST materials, a 27-item survey was also sent requesting feedback on the reports. Questions addressed visual appeal, importance of content, and understandability of data on a scale of 1 being strongly disagreed to 10 strongly agreeing and were derived from previous research (17) but modified to be specific for the Get Fruved reports. RCT site participants were also asked to provide any additional comments they had regarding the report, the PowerPoint, and poster. Feedback was compiled and utilized for changes to be made between the baseline and post-intervention reports. Summative content analysis was used to report compiled feedback comments and quotes (36).

## Results

### Report Building

**Figure 2:**
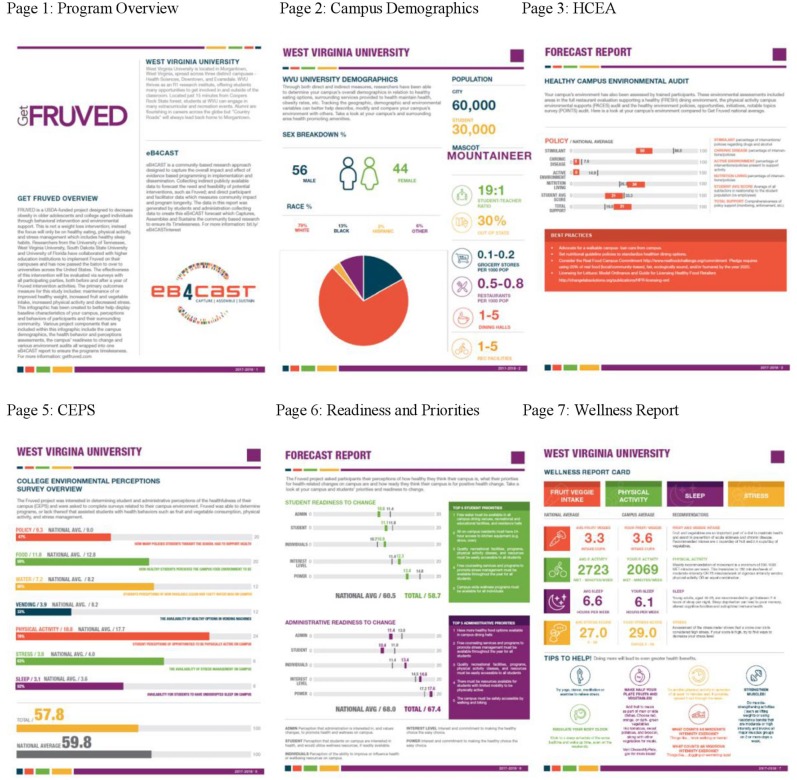
Eight-page sample forecast report for feedback. All pages were finalized after expert feedback and used to gain feedback from Get Fruved Randomized Control Trial sites. Page 1 shows program overview of Get Fruved, eB4CAST, and the university. Page 2 includes campus demographics of the population, sex, race, mascot, student-teacher ratio, out of state population, and the number of grocery stores, restaurants, dining halls, and recreational facilities. Page 3 gives an overview of the sites Healthy Campus Environmental Audit. Page 5 is results of their College Environmental Perceptions Survey. Page 6 is the results of their Student and Administrative priorities and readiness to change. Page 7 is the Wellness Report including results from self-reported tools based on fruit and vegetable consumption, physical activity, perceived stress, and sleep. *Page 4 Recreation and Dining Audit not shown **Page 8 Take Home Message not shown. Figure previously published and has permission to be used in this manuscript from Olfert et al. (16).

Of the 16 requests for graphics assistance, 3 graphic design teams agreed to provide a proposal design to be used to find the best visual reporting system for Get Fruved RCT eB4CAST reports. Weekly meetings with graphic design teams were held from September through December 2017 to set and monitor goals, update progress, and make continuous changes to reports. Three completed infographic reports were finalized in December of 2017. Components of each infographic were compiled into one cohesive and understandable report template. This eight-page template was used to complete initial reports for expert feedback (Figure 2).

### Expert Feedback and Revisions

Prior to the initial dispersion of Forecast reports to RCT sites, feedback data were collected from 14 individuals who are experts in their field and multi-state research team members. To gauge the field, experts were asked their familiarity with the Get Fruved program and the RE-AIM framework on a scale of 1 as not familiar at all to 10 as highly familiar, with a mean result of 9.57 ± 0.76 and 5.44 ± 3.54, respectively. Initial reactions to the report were overwhelmingly positive including statements of “very informative,” “colorful,” “professional,” “appealing,” “comprehensive,” and “thorough.” Two constructive comments from initial reactions included the report being a bit “wordy,” and potentially too “scientific sounding” for the array of intended audiences. Thirteen of the 14 experts provided complete data on all questions. Eleven of the 13 (84.6%) individuals who completed the rating questions rated the Get Fruved infographic, between 1=strongly disagree to 10=strongly agree, as appealing between 8 and 10 (mean of 8.84 ± 1.28) (Table 2). Mean score of 8.08 ± 2.56 was given by experts regarding a clear understanding of the Get Fruved program. Experts scored the infographic being easy to read and providing value overall as 7.92 ± 2.33 and 8.85 ± 2.12, respectively. Regarding if experts believed the community information presented in the infographic was relevant to the Get Fruved program, the average score was 8.07 ± 2.99. Lastly, an average score of 8.46 ± 2.18 was ranked for experts believing these infographics would be helpful to share with community members about the project.

**Table 2:** Review questions and feedback rating from experts.

**Statement**	**Rank (1-10)**
**(*****n*** **=** **13)**	**Mean** **±** **SD**
The get fruved infographic is visually appealing	8.84 ± 1.28
After reading the eB4CAST/get fruved infographic, i have a clear understanding of the get fruved program	8.08 ± 2.56
The infographic was easy to read	8.08 ± 2.56
The infographic provides value	8.85 ± 2.12
The community information presented in the infographic was relevant to the Get Fruved program	8.07 ± 2.99
This infographic would be helpful to share with community members to spread the word about the get fruved program	8.46 ± 2.18
**Ease of understanding data on**	
Healthy campus environmental audit	7.46 ± 2.30
College environmental perceptions survey	7.15 ± 2.82
Student & administrative readiness	7.54 ± 2.93
Student & administrative priorities	8.00 ± 2.71

*Data averaged and reported in mean and standard deviation (SD). Rating of statements were on a scale of 1 being strongly disagree to 10 being strongly agree*.

For overall content of the report, experts were asked to address whether additional information should be included if information should be removed, and if any sections needed to be clarified. Three comments made on overall content included (1) making informational paragraphs more concise and less wordy, (2) to personalize the content more, and (3) to include national average markings on all the tools to allow schools to see where they compare to the average of the schools in Get Fruved. Upon expert feedback, alterations were made to the report to ensure the quality, conciseness, and understandability of the information.

### GetFruved Participant Feedback

A revised and finalized report was sent out to RCT sites (Figure 2). Changes made included averages of the Get Fruved RCT data were added to the individual site data (page 3, 5, and 6; Figure 2) pages, explanations of data and tools were made more clear for each page, demographics were given a full page to personalization including information such as school mascot and a descriptive paragraph overview of the school's history on the front page, and “best practices” suggestions for how to improve campus environment via HCEA scores (Figure 2, page 3).

Of those 62 enrolled Get Fruved RCT sites, *n* = 25 intervention and *n* = 27 control RCT sites completed the baseline data collection and were sent an electronic package of their baseline forecast report (eB4CAST report, PowerPoint and script of data in the report, and a Poster about the program and Student and Administrative Priorities) along with 25 printed copies. Packages were sent to site lead on the project at each RCT site. When receiving their reports, an online Qualtrics (Qualtrics, Provo, UT, USA) survey was included to gather feedback from the sites. From the 57 sites receiving reports and survey, 11 intervention sites and four control sites completed the feedback survey (26.3% response rate). Individual's completing the reports identified themselves as predominately faculty members (*n* = 10; 66.7%), followed by administration (*n* = 4; 26.7%), and one student researcher (*n* = 1; 6.6%). Similar to the expert feedback survey, RCT sites were asked their familiarity with the Get Fruved program and the RE-AIM framework on a scale of 1 as not familiar at all to 10 highly familiar, with a result of 8.20 ± 1.61 and 2.27 ± 2.89, respectively (Table 3).

**Table 3:** Review questions and feedback rating from RCT Sites.

**Statement**	**Rate (1-10)**
**(*****n*** **=** **15)**	**Mean** **±** **SD**
The get fruved infographic is visually appealing	8.50 ± 1.45
After reading the eB4CAST/get fruved infographic, i have a clear understanding of the get fruved program	7.50 ± 2.17
The infographic was easy to read	7.57 ± 1.65
The infographic provides value	8.14 ± 1.35
The community information presented in the infographic was relevant to the get fruved program	7.64 ± 2.41
This infographic would be helpful to share with community members to spread the word about the get fruved program	8.00 ± 1.80
**Ease of understanding data on**	
Healthy campus environmental audit	7.46 ± 2.57
College environmental perceptions survey	7.31 ± 2.46
Student & administrative readiness	7.15 ± 2.70
Student & administrative priorities	8.46 ± 1.56

*Data averaged and reported in mean and standard deviation (SD). Rating of statements were on a scale of 1 being strongly disagree to 10 being strongly agree*.

Twelve of the fourteen respondents who completed the ranking questions provided a score ranging between 8 and 10 indicating that the Get Fruved eB4CAST report was visually appealing, and 8 of the 14 ranked between an 8 and 10 indicating they had a clear understanding of the Get Fruved program after seeing the report. Regarding ease of reading and providing value, site leaders reported an average of 7.57 ± 1.65 and 8.14 ± 1.35, respectively. Lastly, on overall content, participants ranked the community information on the report being relevant to the Get Fruved program as 7.64 ± 2.41 and the report being helpful to share with community members when spreading the word about Get Fruved as an 8.00 ± 1.80.

Qualitative data from open-ended survey questions revealed suggestions for improvement on reports from an RCT site standpoint. Three comments suggesting additional material being added to the reports included (1) more specific information on improving low-scoring areas, (2) stating the benchmarks or total national averages for survey tools, and (3) to add in school logos on the reports. We asked participants to list 3–5 descriptions of the report overall. The vast majority stated phrases such as “organized,” “professional,” “informative,” “like having the report in three separate forms,” and “thankful to receive data in a cohesive report.” Participants noted the following negatives about the report: not clear how point systems work, needing benchmarks, and that the report took a long time to read.

For each section of the report, participants were asked to identify any negatives for each respective section: HCEA, CEPS, Priorities, and Readiness. For the four sections, four of the 15 participants suggested to clarification of the scales of the tools used, to make clearer definitions of the subscales, and to provide a copy of the raw questions in a document for reference on variables used in each scale.

## Discussion

The present study highlights respondents' general favorability of eB4CAST as a dissemination tool for effective communication for data from the Get Fruved study. Experts and community members found the tool to be visually appealing, easy to read, and a helpful tool to share with the community. Participants also rated the tool favorably for all areas including visual appearance, readability, relevance, and helpfulness to the community. Overall, results were in favor of using the eB4CAST tool and highlight the potential role that this dissemination tool could play in public health nutrition research. Within Get Fruved specifically, eB4CAST infographics provided effective communication processes by providing concise information in a short time. This quick and clear communication is beneficial when explaining programs to those individuals whom buy-in is needed or whom you want to show the programmatic efforts. This work is promising for the future of dissemination research and may overcome some of the barriers seen in previous literature such as time, cost, communication, and appeal by providing a simple, easy-to-read, visual infographic that is sent to them electronically. eB4CAST incorporates previous frameworks (37–39) and builds an effective tool through *capturing* community data and impact, *assembling* these data into a visually appealing infographic to disseminate for program *sustainability* and *timelessness* (16).

eB4CAST is also flexible and can be modified to reflect a community's needs. Each community can control which data they highlight in their reports, and because of this, eB4CAST can provide ownership to each community's dataset and report (16). the eB4CAST tool evolved from use in a previous study for the dissemination of a childhood obesity prevention program, iCook 4-H (16, 17, 40). As observed with the Get Fruved study, reviewers of the iCook eB4CAST reports found the infographics to be favorable in portraying results of the study, its importance, and representation of the project (17). iCook participants stated that the reports would be useful in overcoming barriers to dissemination, improving communication, and showing program impact (17).

Using CBPR principles in public health nutrition can increase the likelihood of a program sustaining itself by engaging the community (9, 41). With engagement comes the responsibility to effectively relay study outcomes to the community, which is not an easy task based on the research-practice gap issue in the field (42). By developing a tool to improve the communication between researchers and the community, we aim to bridge this gap and disseminate programs in findings sooner into the public sector. Utilizing the eB4CAST infographic reports may help researchers communicate with their communities and provide a successful method of dissemination. Infographics can capture attention and provide information through a minimal amount of wording, and by leveraging our brains' most dominant capacity for learning—visual aid (43).

While feedback was positive in this study among Get Fruved eB4CAST limitations should also be noted. Because the eB4CAST infographic was used solely here with the Get Fruved study and previously with iCook 4-H, it can not necessarily be translated across the board to other community-based programs. However, throughout future work, we aim to streamline the process and make it feasible for use across various programs. The survey used to evaluate the eB4CAST report is not validated which adds additional limitation but was modified from the tool used previously with the iCook 4-H study to specifically address the Get Fruved eB4CAST report (17). Another limitation to note is that the content experts and researchers who were asked to provide feedback in this study were familiar with the Get Fruved program and could have influenced their perception of the readability of the infographic and thus, social desirability bias (44, 45). These experts were also more likely to understand the importance of using the eB4CAST infographics. Further, comparison of expert and RCT site perceptions was not possible since expert feedback was used to make revisions to the eB4CAST reports and thus, the report shown to experts and RCT sites differed. The response rate to feedback was lower than researchers had hoped, specifically among control sites; thus, caution should be taken with the positive results and limited suggestions for report improvement. This lack of participation from the control sites may be due to lack of investment as they were not engaged in the GetFruved content on campus and therefore less likely to understand the value of an eB4CAST report to showcase outcomes and promote dissemination to campus stakeholders. It is unclear whether those individuals who didn't complete the survey would have felt positive or negative about the layout and content. In order to conclude that the reports are well-received and understood by a broader population, more testing should be done in the future with community members, program participants, and outside personnel who are unfamiliar with the programs. Specifically, control sites should be targeted to ensure usefulness in RCT settings.

In future work in the area of D&I methods, researchers should utilize approaches of media avenues for dissemination, such as infographics. The use of infographics has been shown previously to enhance readership of research outcomes along with avenues such as social media and podcasts (18–20, 46). However, there is limited understanding of using research sites' program data to form personalized infographics. For the Get Fruved project, professionals and community members were able to be both research participants and provide feedback as infographics of research outcomes were developed. However, looking into the ways they utilized the information and infographics on their campus for sustainability is of interest. The aim of these reports is to assist programming in longevity. As these reports are personalized to each site and give them feedback on their data findings, it is the hope that a tangible representation of a program will lead to father impact of these programs. Future work should examine the capacity with which members utilize their infographics, as well as the sustainability of these tools and the programs themselves. As we provide a tool for communication, measuring its reach is also noteworthy to capture.

### Implications for Research and Practice

eB4CAST has been showed to be useful and favorable in two CBPR interventions in large scale populations. Further usage of this tool is warranted to aid in lessening the research-practice gap through cost-effective, clear, communication of programs and their outcomes. Utilizing visually appealing and concise communication can be key to provide feedback to community members and research stakeholders to evaluate the effectiveness of the interventions ran. For future research and practice tools like this can be utilized to extend interest of funders, administration or communities to further research.

## Data Availability Statement

The raw data supporting the conclusions of this article will be made available by the authors, without undue reservation, to any qualified researcher.

## Ethics Statement

The multi-state umbrella Institutional Review Board at the University of Tennessee, Knoxville approved all aspects of the study for all researchers developing eB4CAST and Get Fruved RCT sites (IRB approval #UTK IRB-14-09366 B-XP). The University of Florida IRB approved the same strategies for activities at the University of Florida (IRB approval #2014-U-0547 FRUVED). The study was conducted in accordance with the Declaration of Helsinki and all participants provided written consent to participate by signing an IRB approved informed consent form. Verbal consent was received from each participant.

## Author Contributions

Funding acquisition by MO, SC, WZ, AM, KK, TK, OB, LF-C, AW, GG, KS, CB-B, and TH. All authors participated in design and data collection. Analyses completed by MB. Draft of the manuscript prepared by MO, MB, RH, and RW. All authors edited and approved the final manuscript.

## Conflict of Interest

The authors declare that the research was conducted in the absence of any commercial or financial relationships that could be construed as a potential conflict of interest.
